# Activating Lattice Oxygen in Perovskite Oxide by B‐Site Cation Doping for Modulated Stability and Activity at Elevated Temperatures

**DOI:** 10.1002/advs.202102713

**Published:** 2021-10-18

**Authors:** Huijun Chen, Chaesung Lim, Mengzhen Zhou, Zuyun He, Xiang Sun, Xiaobao Li, Yongjian Ye, Ting Tan, Hui Zhang, Chenghao Yang, Jeong Woo Han, Yan Chen

**Affiliations:** ^1^ School of Environment and Energy State Key Laboratory of Pulp and Paper Engineering South China University of Technology Guangzhou Guangdong 510006 China; ^2^ Department of Chemical Engineering Pohang University of Science and Technology Pohang Gyeongbuk 37673 Republic of Korea; ^3^ State Key Laboratory of Functional Materials for Informatics Shanghai Institute of Microsystem and Information Technology Chinese Academy of Sciences Shanghai 200050 China

**Keywords:** cation doping, Fe—O octahedra, metal—oxygen bond, oxygen vacancy, perovskite oxide

## Abstract

Doping perovskite oxide with different cations is used to improve its electro‐catalytic performance for various energy and environment devices. In this work, an activated lattice oxygen activity in Pr_0.4_Sr_0.6_Co*_x_* Fe_0.9−_ *_x_* Nb_0.1_O_3−_ *_*δ*_* (PSCxFN, *x* = 0, 0.2, 0.7) thin film model system by B‐site cation doping is reported. As Co doping level increases, PSCxFN thin films exhibit higher concentration of oxygen vacancies ($V_{o}^{••}$) as revealed by X‐ray diffraction and synchrotron‐based X‐ray photoelectron spectroscopy. Density functional theory calculation results suggest that Co doping leads to more distortion in Fe—O octahedra and weaker metal—oxygen bonds caused by the increase of antibonding state, thereby lowering $V_{o}^{••}$ formation energy. As a consequence, PSCxFN thin film with higher Co‐doping level presents larger amount of exsolved particles on the surface. Both the facilitated $V_{o}^{••}$ formation and B‐site cation exsolution lead to the enhanced hydrogen oxidation reaction (HOR) activity. Excessive Co doping until 70%, nevertheless, results in partial decomposition of thin film and degrades the stability. Pr_0.4_S_r0.6_(Co_0.2_Fe_0.7_Nb_0.1_)O_3_ with moderate Co doping level displays both good HOR activity and stability. This work clarifies the critical role of B‐site cation doping in determining the $V_{o}^{••}$ formation process, the surface activity, and structure stability of perovskite oxides.

## Graphical abstract

An activated lattice oxygen activity in Pr_0.4_Sr_0.6_Co*_x_* Fe_0_._9−_ *_x_* Nb_0.1_O_3−_ *_*δ*_* (PSCxFN, *x* = 0, 0.2, 0.7) thin film model system by B‐site cation doping is reported. Co doping in PSCxFN facilitates oxygen vacancy formation and promotes B‐site cation exsolution, thereby enhances the electrochemical performance at elevated temperatures. Excessive Co doping until 70%, nevertheless, results in partial decomposition of the materials and degrades the stability.

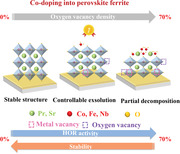

## 1 Introduction

Because of their unique structure stability, redox reversibility, high catalytic activity, and low price, perovskite oxides ABO_3_ have been widely used as catalysts for various reactions in energy and environment devices, such as solid oxide fuel cell/electrolysis cell (SOFC/SOEC),^[^ ^1^ ^]^ solar‐thermal H_2_O/CO_2_ splitting,^[^ ^2^ ^]^ and volatile organic compounds oxidation.^[^ ^3^ ^]^ The catalytic activity of perovskite oxides was found to be largely determined by the characteristics of lattice oxygen, including the oxygen nonstoichiometry (oxygen vacancy concentration),^[^ ^4^ ^]^ asymmetry of oxygen‐transition metal octahedra,^[^ ^5^ ^]^ metal—oxygen covalence,^[^ ^6^ ^]^ etc. To promote the surface reactions, great efforts have been devoted to tune the lattice oxygen properties via approaches such as introducing elastic strain,^[^ ^7^ ^]^ constructing hetero‐interface,^[^ ^8^ ^]^ nanostructure engineering,^[^ ^9^ ^]^ and cation doping.^[^ ^10^ ^]^ Among all these approaches, cation doping is most widely used in literatures due to its simple process and wide range of selections. Particularly, doping cations with lower valence in A‐site lattice is a commonly used strategy to introduce oxygen vacancies ($V_{o}^{••}$) into the lattice. Representative example is to replace La^3+^ with Sr^2+^ in LaMO_3_ (M represents transition metal cations).^[^ ^11^ ^]^ Cation dopant with lower oxidation states at A‐site of perovskite oxides were reported to oxidize B‐site cation^[^ ^12^ ^]^ and weaken the metal—oxygen bond.^[^ ^13^ ^]^ Accompanied by such changes, oxygen vacancies form in the lattice to compensate the charge and maintain the electrical neutralization of material system.^[^ ^14^ ^]^ In addition to A‐site doping, replacing B‐site cations with higher reducible metal elements in perovskite lattice was also reported to promote the lattice oxygen activity.^[^ ^15^ ^]^ The mechanism of such promotion, nevertheless, is less revealed and requires systematical investigation.

For various applications such as fuel electrodes for SOFC/SOEC, the perovskite catalysts need to remain stable in reducing environment at elevated temperatures. Although perovskite oxides such as Sr_2_Fe_1.5_Mo_0.5_O_6−_ *_*δ*_*,^[^ ^16^ ^]^ La_0.75_Sr_0.25_Cr_0.5_Mn_0.5_O_3_,^[^ ^17^ ^]^ and PrBaMn_2_O_5+_ *_*δ*_* ^[^ ^18^ ^]^ were reported to be stable in hydrogen/hydrocarbon gas environment, their catalytic performances cannot meet the practical requirement of the devices.^[^ ^19^ ^]^ Interestingly, it was reported that by doping certain cations, particularly reducible transition metals or noble metals, into the B‐site lattice, metal nanoparticles can exsolve to the surface after reduced in high temperature, while the matrix can either maintain perovskite structure or go through phase transition.^[^ ^20^ ^]^ Although the structures of these perovskite oxides became seemly less stable, the obtained oxide matrixes with exsolved nanoparticles exhibited strongly enhanced catalytic activities.^[^ ^21^ ^]^ Myung et al. reported that perovskite La_0.43_Ca_0.37_Ni_0.06_Ti_0.94_O_3−_ *_*γ*_* with exsolved Ni nanoparticles presented high performance for hydrogen oxidation reaction with outstanding long‐term stability.^[^ ^1a^ ^]^ Opitz et al. found that La_0.6_Sr_0.4_FeO_3−_ *_*δ*_* with formation of Fe nanoparticles on surface displayed strongly enhanced activity for electrochemical water splitting.^[^ ^22^ ^]^ All these pioneering works have demonstrated exsolution as an effective approach to improve the catalytic activity of perovskite oxides. The fundamental understanding about the driving force for cation exsolution and factors that determined the kinetics of cation migration, nevertheless, are still ill‐defined and even controversial in certain cases.

There have been many previous works investigating the exsolution behavior of perovskite oxides with different B‐site cation doping.^[^ ^4^, ^23^ ^]^ Kwon et al.^[^ ^10a^ ^]^ reported that the presence of $V_{o}^{••}$ facilitated the segregation of reducible Co, Ni to the surface by co‐segregation process, i.e., $V_{o}^{••}$ and cations migrated together, in layer perovskite oxide PrBaMn_1.7_T_0.3_O_5+_ *_*δ*_* (T = Mn, Co, Ni, and Fe). Lv et al. found Co dopant on double perovskite Sr_2_Fe_1.35_Mo_0.45_Co_0.2_O_6−_ *_*δ*_* led to the formation of cation vacancies and $V_{o}^{••}$, which promoted Fe exsolution.^[^ ^24^ ^]^ All these works suggest that $V_{o}^{••}$ formation process in perovskite oxides critically impacts the exsolution behavior. Therefore, revealing the impact of B‐site cation doping on $V_{o}^{••}$ formation and the lattice stability of perovskite oxides are critical for understanding the mechanism of exsolution.

In this study, we investigated systematically the impact of Co doping on the $V_{o}^{••}$ formation process and the lattice stability of Pr_0.4_Sr_0.6_Co*_x_* Fe_0.9−_ *_x_* Nb_0.1_O_3−_ *_*δ*_* (PSCxFN, *x* = 0, 0.2, 0.7) by the combination of experimental and computational approaches. Highly textured PSCxFN thin films prepared by pulsed laser deposition (PLD) on yttrium‐stabilized ZrO_2_ (YSZ) substrate were used as the model system to avoid the complication arising from microstructures. High‐resolution X‐ray diffraction (HRXRD) and synchrotron‐based ambient pressure X‐ray photoelectron spectroscopy (AP‐XPS) results suggested that Co doping strongly facilitated $V_{o}^{••}$ formation in PSCxFN film when reduced in hydrogen gas environment. Consistently, the $V_{o}^{••}$ formation energy calculated by density functional theory (DFT) was found to be decreased with Co doping level. DFT results further showed that Co doping in PSCxFN phase effectively reduced the metal—oxygen bond strength by increase of metal—oxygen antibonding state, which facilitated $V_{o}^{••}$ formation. The local Fe—O octrahedral asymmetry was greatly impacted by the Co doping, which also likely modified the $V_{o}^{••}$ formation process. Accompanied with the higher concentration of $V_{o}^{••}$, PSCxFN with higher Co content exhibited lower stability with more Co/Fe‐enriched exsolution phase on the surface. Due to the competing effects of activating oxygen activity and decreasing structure stability, the perovskite thin film with highest Co content exhibited the highest initial hydrogen oxidation reaction activity but poorest long‐term stability. Our results clarify the critical role of B‐site doping on the oxygen activities and stability of perovskite oxides. Such mechanistic understanding can help guide the rational design of perovskite oxide catalysts for high‐temperature (electro)chemical devices for energy and environment applications.

## 2 Results and Discussion

### 2.1 Chemical Expansion Induced by Oxygen Vacancy Formation

**Figure 1:**
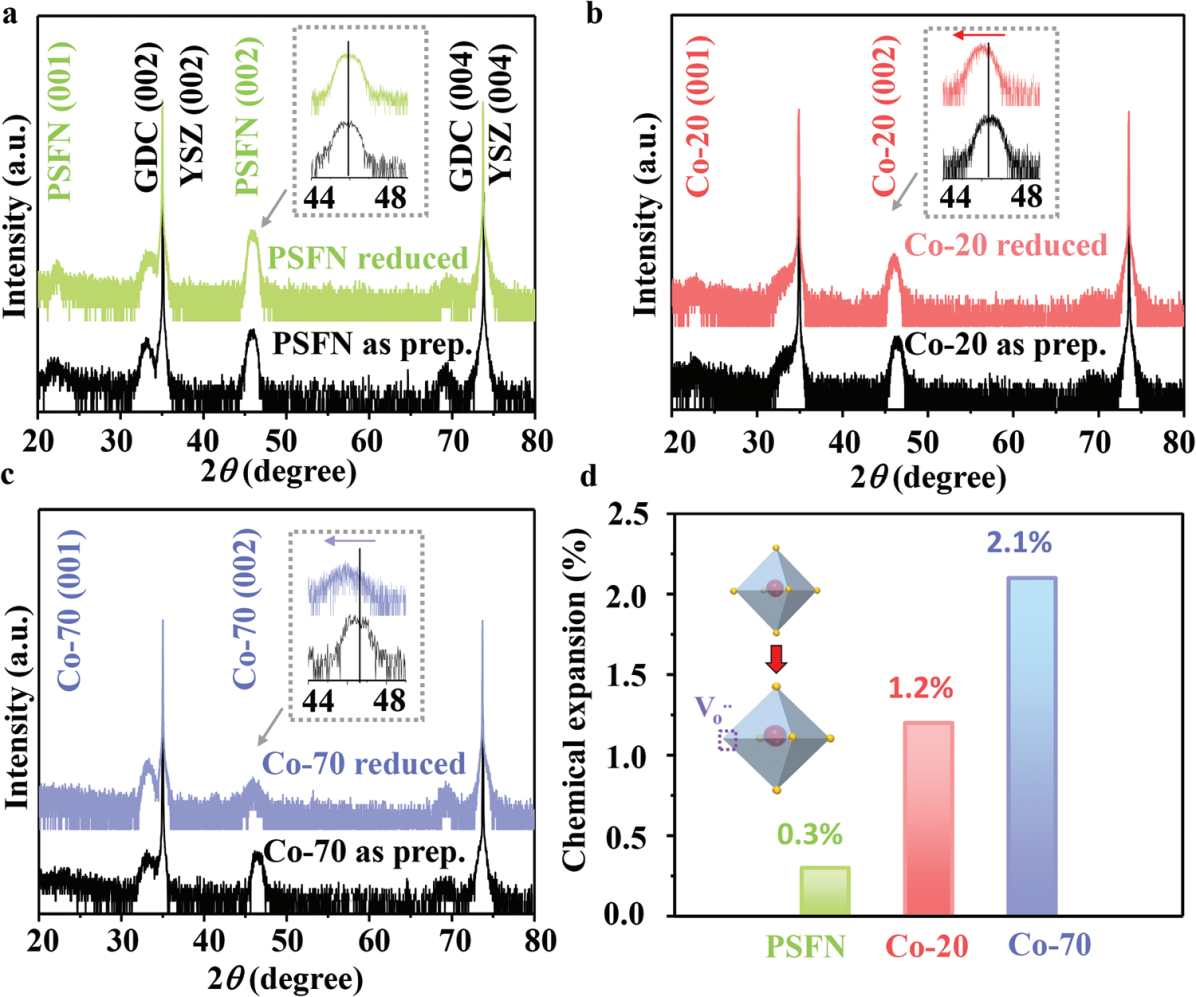
HRXRD patterns of a) PSFN, b) Co‐20, and c) Co‐70 in as prep. condition (black lines) and after reduced at 650 °C for 2 h in pure H_2_. The insert is enlarged image for characteristic peaks with the range from 44° to 48°. d) The corresponding chemical expansion of thin films from HRXRD results in (a).

Pr_0.4_Sr_0.6_Co*_x_* Fe_0.9−_ *_x_* Nb_0.1_O_3−_ *_*δ*_* (PSCxFN, *x* = 0, 0.2, 0.7) thin films with different Co concentrations at B‐site were grown on single‐crystal YSZ (001) substrate by PLD. A thin layer of gadolinium‐doped ceria (GDC) was deposited as the buffer layer between PSCxFN thin film and YSZ electrolyte, which can effectively prevent the undesired interfacial reaction between the electrodes and electrolyte. The obtained thin films were denoted as PSFN, Co‐20, Co‐70 for *x* equals to 0, 0.2, and 0.7 in the following context, respectively. HRXRD results showed that all the films were highly textured with (001) orientation (**Figure** **1**). The thicknesses of the PSCxFN thin films were close to 60 nm (Figure S1, Supporting Information).

The crystal structures of PSCxFN thin films in the as‐prepared state and after thermal reduction in pure H_2_ at 650 °C for 2 h were compared. The Pr_0.4_Sr_0.6_Fe_0.9_Nb_0.1_O_3_ (PSFN) exhibited a stable ABO_3_ perovskite structure without obvious film peak shift after thermal reduction, suggesting a negligible change in the lattice parameters upon reduction (Figure 1a). For the Co‐20 sample, the peak position of the film shifted toward lower 2*θ* value (red arrow in Figure 1b), which corresponded to 1.2% lattice expansion (Figure 1d), while the peaks for substrate and the GDC buffer layer remained unchanged. With increasing Co doping to 70%, the film peak further shifted toward lower 2*θ* value (blue arrow in Figure 1c), corresponding to the largest lattice expansion of 2.1% (Figure 1d). When metal oxide became reduced, the formation of $V_{o}^{••}$ was normally accompanied by the change of metal valence states, and both effects can change the size of unit cell.^[^ ^25^ ^]^ Normally, perovskite oxide exhibited positive chemical expansion upon the $V_{o}^{••}$ formation due to the much larger radius for the transition metal ion with lower valence states.^[^ ^8^, ^26^ ^]^ The larger lattice expansion for the PSCxFN films with higher Co doping level implies more $V_{o}^{••}$ upon thermal reduction.

It is important to note that the XRD peak intensity of PSFN and Co‐20 did not show noticeable changes after reduction, whereas the XRD peak intensity of Co‐70 decreased dramatically (Figure 1c). Such decrease of peak intensity of Co‐70 thin film is likely due to its less stable crystal structure, which will be further discussed in the following context.

### 2.2 Oxygen Chemical Environment Probed by Synchrotron‐Based AP‐XPS

**Figure 2:**
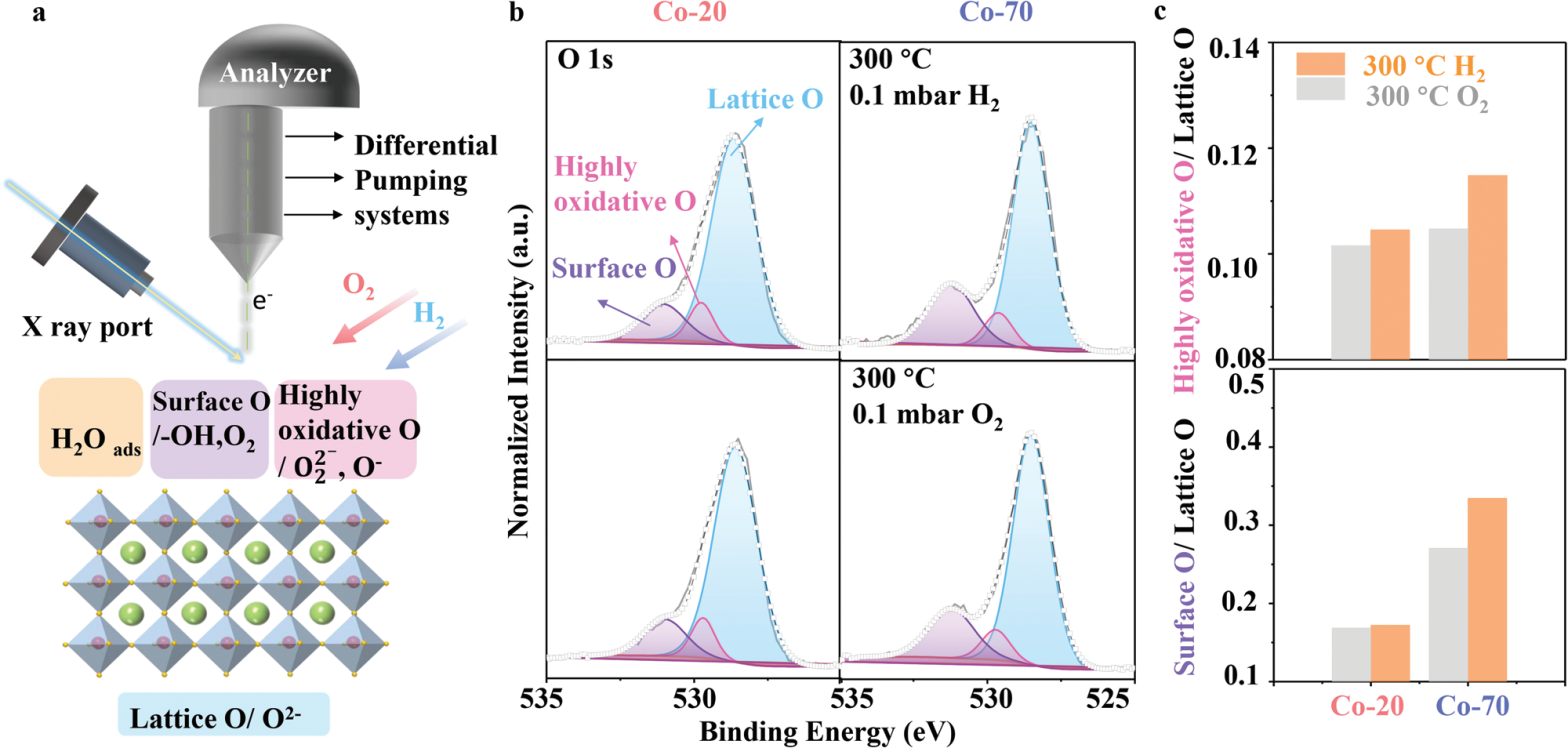
a) Schematic diagram about the working principle of the AP‐XPS setup for in situ measurement in different gas environments. The bottom figure shows the different surface oxygen species such as H_2_O_ads_, surface O, highly oxidative O, and lattice O on perovskite oxides surface. b) O 1s spectra of Co‐20 (left) and Co‐70 (right) films were collected at 300 °C in 0.1 mbar O_2_ (bottom) and 0.1 mbar H_2_ (top), respectively. The spectra were fitted by different oxygen components, including surface O (purple), highly oxidative O (pink), lattice O (blue). c) Comparison of highly oxidative O/lattice O ratio and surface O/lattice O ratio for Co‐20 and Co‐70 samples at different conditions.

Because of oxygen‐containing contaminates on the surface, it is difficult to use ex situ XPS measurement to probe the chemical environment of oxygen on transition metal oxides surface. To reveal the correlation between oxygen chemical environment and Co doping level in PSCxFN film, synchrotron‐based AP‐XPS (**Figure** **2**a) was used to characterize Co‐20 and Co‐70 thin films at 300 °C in 0.1 mbar oxygen and 0.1 mbar hydrogen. The relatively low temperature of 300^ ^°C ensured that the cations were not mobile, while oxygen in the lattice can exchange with that in the environment. As confirmed by AP‐XPS and atomic force microscope results, the surface cation compositions were not changed (Figure S2, Supporting Information) and the surfaces remained smooth (Figure S3, Supporting Information) after in situ measurement. Before the in situ measurement, all thin films were cleaned by heating in 0.1 mbar O_2_ at 300 °C for 1 h to ensure that the C 1s peak located at 284.6 eV and O 1s peak of H_2_O_ads_ located at 532.5 eV were not detected (Figure S4, Supporting Information and Figure 2b).

The O 1s spectra of Co‐20 and Co‐70 films collected at 300 °C in 0.1 mbar O_2_ and 0.1 mbar H_2_ are shown in Figure 2b, which were divided into three peaks for different oxygen species: surface‐adsorbed oxygen (surface O, like ‐OH) at 530.9 eV, highly oxidative oxygen relative to detective oxygen (O^−^, $O_{2}^{2-}$) at 529.6 eV, lattice oxygen (O^2−^) at 528.5 eV.^[^ ^27^ ^]^ The highly oxidative O was reported to be closely correlated to surface $V_{o}^{••}$.^[^ ^27^, ^28^ ^]^ For both Co‐20 and Co‐70, the highly oxidative O/lattice O ratio were higher in 0.1 mbar H_2_ than that in 0.1 mbar O_2_ (Figure 2b,c), suggesting the formation of extra $V_{o}^{••}\;$when switching from O_2_ to H_2_ gas atmosphere. The increase of highly oxidative O/lattice O ratio for Co‐70 is more pronounced than that for the Co‐20 sample. This result indicates that it is easier to form $V_{o}^{••}$ in Co‐70 than that in Co‐20.

The presence of oxygen defects was reported to facilitate the formation of other oxygen‐containing species such as ‐OH group on the surface.^[^ ^27^, ^29^ ^]^ In both O_2_ and H_2_ environment, the Co‐70 sample exhibited higher content of highly oxidative O compared with the Co‐20, suggesting larger amount of $V_{o}^{••}$ in the Co‐70 sample. As a result, the surface O/lattice O ratio for the Co‐70 is also higher than that for the Co‐20 (Figure 2c). After annealed in H_2_, the surface O/lattice O ratio for the Co‐70 pronouncedly increased, while the surface O/lattice O ratio for the Co‐20 remained unchanged. Such difference is likely due to the higher reactivity of Co‐70 surface to hydrogen than that of Co‐20, leading to the formation of other oxygen species such as ‐OH group.

### 2.3 Oxygen Vacancy Formation Energy and Electronic Structure

To reveal the role of Co in the formation of $V_{o}^{••}$, we carried out DFT calculation to determine $V_{o}^{••}$ formation energy (*E*_f_vac_), electronic structure and the octahedral distortion in Pr_0.5_Sr_0.5_Co*_x_* Fe_1−_ *_x_* O_3_ system (PSCxF, *x* = 0, 0.25, 0.5, 0.75). The investigated compositions were denoted as PSF, PSCF25, PSCF50, PSCF75, respectively, in the following context of this section.

**Figure 3:**
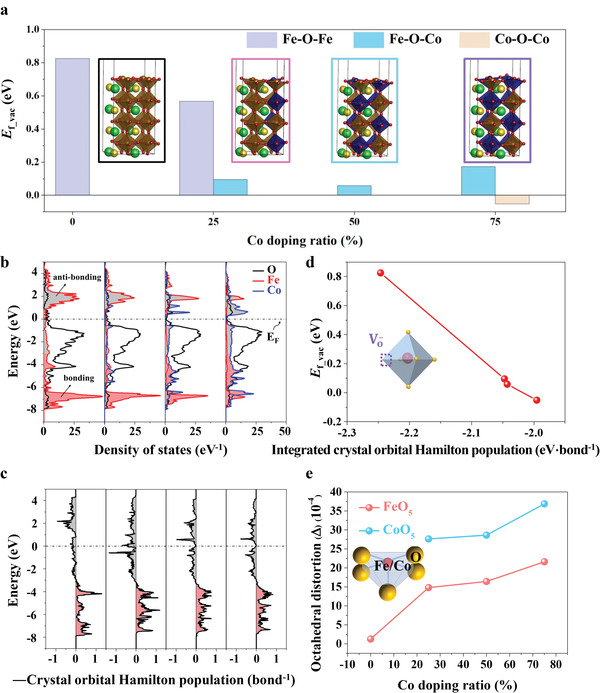
a) Surface oxygen vacancy formation energies (*E*_f_vac_) and b) projected DOS of bulk PSCxF models with different Co doping ratios were compared. The *x* equals to 0, 0.25, 0.5, and 0.75. The insert images in (a) were the thin‐film models used in DFT calculations. c) Crystal orbital Hamilton populations (‐COHP) plots of Fe—O bond in bulk PSF and Co—O bond in bulk PSCxF. Gray area (‐COHP: minus) is the antibonding area and red area (‐CPHP: plus) is the bonding area. d) Lowest *E*_f_vac_ in PSCxF as a function of the integrated COHP (ICOHP) of Fe—O bond in PSF and Co—O bond in PSCxF obtained from DFT calculations. The inset figure in the left of (d) corresponds to oxygen vacancy formation of MO_6_ octahedron (M: Fe, Co). e) The octahedral distortion of half‐octahedron (FeO_5_ and CoO_5_) for the thin films was calculated. The half‐octahedron model was shown as the inserted image.

The *E*_f_vac_ at different sites for PSF, PSCF25, PSCF50, PSCF75 were determined by using Equation (1). As shown in **Figure** **3**a, *E*_f_vac_ at Fe‐O‐Fe, Fe‐O‐Co/Fe‐O‐Fe, Fe‐O‐Co, Fe‐O‐Co/Co‐O‐Co for PSF, PSCF25, PSCF50, and PSCF75 was compared, respectively. Among all calculated sites on PSCxF surface, PSF exhibited maximum *E*_f_vac_ (0.83 eV), indicating that PSF tends to form low concentration of $V_{o}^{••}$ on surface. When Co was doped in bulk, the *E*_f_vac_ of PSCF25 at Fe‐O‐Co site (0.10 eV) was lower than that at Fe‐O‐Fe site (0.57 eV). Similar decreased *E*_f_vac_ was also observed in PSCF75, while the *E*_f_vac_ at Co‐O‐Co site was the lowest (−0.05 eV). Based on *E*_f_vac_ comparison at different sites in the individual model, we found the *E*_f_vac_ was lower at the sites where there were more Co—O bonds (PSCF25: Fe‐O‐Fe > Fe‐O‐Co, PSCF75: Fe‐O‐Co > Co‐O‐Co). Therefore, the concentration of $V_{o}^{••}$ is predicted to increase as Co ratio and Co—O bonds increase. These results are consistent with the promotion of $V_{o}^{••}$ formation by Co doping, which we observed experimentally above.

The density of states (DOS) for PSCxF was further calculated to evaluate the impact of Co doping on the electronic structure (Figure 3b). The accurate bonding and antibonding states were calculated by crystal‐orbital Hamilton population (COHP; Figure 3c). COHP is the product of the DOS and the overlap Hamiltonian element. The negative and positive values of ‐COHP correspond to the antibonding and bonding states of Fe—O (for PSF) and Co—O bond (for PSCxF), respectively.^[^ ^30^ ^]^ For PSF, the O 2p band was mainly overlapped with Fe 3d bonding state (Fe DOS peak and ‐COHP peak whose energy level was less than −3 eV, Figure 3b,c).^[^ ^31^ ^]^ The antibonding state (Fe DOS peak and ‐COHP peak whose energy level is higher than 0 eV, Figure 3b,c) was almost unoccupied state in PSF because its energy level was above the Fermi level. This result indicates that PSF has strong Fe—O bonding,^[^ ^31a,b^ ^]^ which may result in decreased concentration of free electrons and sluggish process of electron transfer.^[^ ^32^ ^]^ After replacing Fe with Co in PSCxF, the antibonding state of Co 3d band (Co DOS peak and ‐COHP peak whose energy level is higher than −2 eV, Figure 3b,c) appeared under Fermi level. The region where O 2p band was overlapped with Co 3d antibonding state was partially occupied state under Fermi level. This indicates that the Co—O bonding of Co‐doping thin film models is weaker than Fe—O bonding of PSF.^[^ ^31a,b^ ^]^

In order to analyze the electronic structure of PSCxF quantitatively, the integral of COHP up to the Fermi level (integrated COHP, ICOHP) was calculated from DOS result. ICOHP can be used to compare the metal—oxygen bond strength.^[^ ^31c^ ^]^ ICOHP increased as Co ratio increased, following the order of PSF (−2.25 eV) > PSCF25 (−2.05 eV) > PSCF50 (−2.04 eV) > PSCF75 (−2.00 eV). To show the relationship between band structure and $V_{o}^{••}$ formation, the COHP of Co—O bonds was used for Co‐doping thin films and the lowest *E*_f_vac_ was used for PSCF25 and PSCF75. As a result, the linear relationship could be found between *E*_f_vac_ and ICOHP (Figure 3d). ICOHP in PSF was much lower than that in Co‐doping thin films. It showed that the downshift of antibonding state was the main reason of smaller $V_{o}^{••}$ formation energy with more increased Co doping.

The DOS analysis showed that Co—O bonds of Co‐doping thin films were weaker than Fe—O bond of PSF. However, the *E*_f_vac_ of Fe‐O‐Fe site of PSCF25 (0.57 eV) was also much lower than the *E*_f_vac_ at the same site of PSF (0.83 eV). To find the other factor that makes the difference on *E*_f_vac_, the octahedral distortion of surface layer was evaluated with Co doping ratios (Figure 3e). Because the thin‐film models were BO_2_‐terminated, the octahedral structures were sliced to half‐octahedron (CoO_5_ and FeO_5_). CoO_5_ octahedral distortion (27.62 × 10^−4^) in PSCF25, which was defined by Equation (2) in the Experimental Section, was higher than FeO_5_ octahedral distortion (1.26 × 10^−4^) in PSF. It indicates that the Co doping weakens the metal—O bonds in perovskites, which supports the above‐mentioned DOS results. However, increasing the Co doping has a slight effect on CoO_5_ octahedral distortion, indicating the change of CoO_5_ octahedral distortion is not the main reason for lower $V_{o}^{••}$ as increased Co doping. It was reported that perovskite ferrites with more distorted FeO_6_ octahedral exhibited lower $V_{o}^{••}$ formation energy.^[^ ^5^ ^]^ In this work, the FeO_5_ octahedral distortion of PSCF25 (FeO_5_: 14.80 × 10^−4^) was higher than that of PSF (FeO_5_: 1.26 × 10^−4^). FeO_5_ octahedral distortion of PSCF50 and PSCF75 was also higher than that of PSCF25. It showed that Co doping increased the geometrical reducibility as well as the electronical reducibility. The geometrical reducibility affects Fe‐O‐Fe site of PSCxF, and as a result, the *E*_f_vac_ at Fe‐O‐Fe site of PSCF25 can be lower than the *E*_f_vac_ at the same site of PSF.

DFT studies showed that Co‐doping thin films displayed lower *E*_f_vac_ than that in PSF due to weaker metal—O bonds, decreased band center difference and more octahedral distortion of FeO_5_. Therefore, PSCxFN thin films with higher Co content are likely to form more $V_{o}^{••}$ than that in PSFN. These results are consistent with HRXRD peak shift toward lower 2*θ* value (Figure 1). Our DFT results not only reveal the easier formation of surface $V_{o}^{••}$ as a function of Co doping, but also uncover the possible mechanism of electronic structure on such promotion of $V_{o}^{••}$ formation.

In addition to our results, it was also reported that Co‐doping can effectively promote the oxygen vacancy formation process in other perovskite oxide systems, such as Co‐doped Sr_2_Fe_1.5_Mo_0.5_O_6−_ *_*δ*_*,^[^ ^24^ ^]^ Co‐doped Pr_0.5_Ba_0.5_MnO*_x_*.^[^ ^33^ ^]^ We believed that the mechanistic understanding we obtained in this work about impact of Co doping on oxygen activity can be applicable to these perovskite oxide systems. The methodology used in this work can also be used for understanding the effect of other B‐site dopants on oxygen activity of perovskite oxides.

### 2.4 Surface Cation Exsolution

In the previous section, we have demonstrated that Co doping can effectively promote $V_{o}^{••}$ formation. More $V_{o}^{••}$ were reported to facilitate the B‐site cation exsolution toward surface.^[^ ^10a^ ^]^ Upon exposure to a reducing atmosphere, mass loss of oxygen is more likely to happen on Co‐doping thin‐films surface. Subsequently larger *p*O_2_ gradient between the perovskite lattice and external environment can potentially lead to lattice reduction and further cation exsolution.^[^ ^1a^ ^]^ Therefore, in this section we further evaluated the impact of Co doping on stability of the lattice by investigating the surface exsolution behaviors.

**Figure 4:**
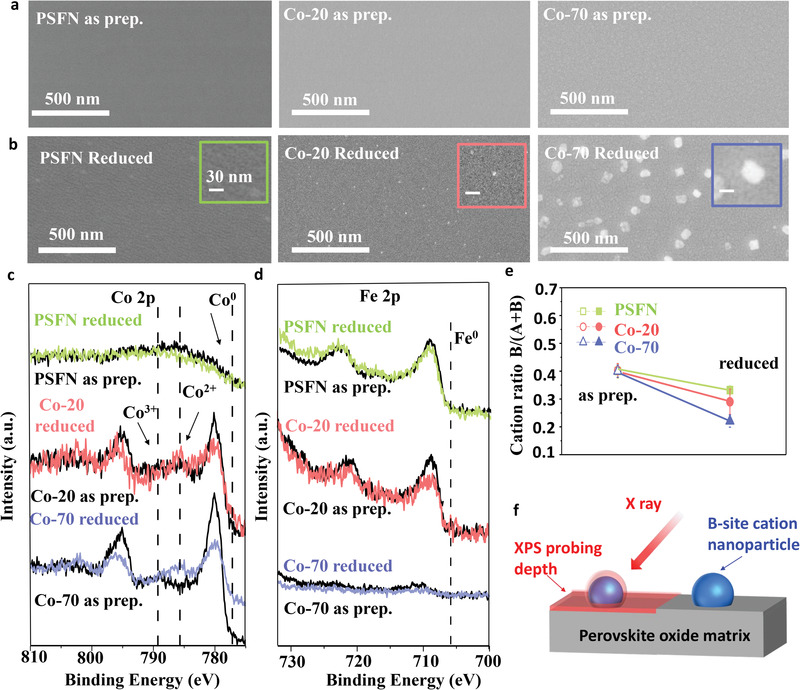
SEM images of PSCxFN thin films in a) as‐prepared states and b) after reduced in pure H_2_ at 650 °C for 2 h. c) Co 2p and d) Fe 2p XPS spectra for PSCxFN thin films in their as‐prepared state (black lines) and after hydrogen reduction (green line for PSFN, red line for Co‐20, blue line for Co‐70). e) Comparison of B‐site cation ratios for PSCxFN thin films before and after reduction. f) Schematics showed the smaller probing depths of XPS measurement in comparison to the size of exsolved particles, which lead to the seemly lower B‐site (Fe/Co) contents on sample surface after reduction.

The surface morphology of PSCxFN thin films in the as‐prepared state and after thermal reduction in pure H_2_ at 650 °C for 2 h was detected by scanning electron microscope (SEM), as shown in **Figure** **4**a. All the films exhibited smooth surface without any secondary phase in the as‐prepared state. After thermal reduction, only scattered particles were observed on the PSFN surface and most regions remained smooth (Figure 4b). In contrast, nanoparticles with the size of ≈10 nm appeared on the Co‐20 surface after reduction. Further increasing Co doping in B‐site to 70% led to the formation of larger nanoparticles formation with the size of ≈50 nm on its surface after reduction. Such morphology evolution suggests that the PSCxFN thin films became unstable when Co doping level increased.

*Ex‐situ* XPS measurements were carried out on the thin films before and after thermal reduction to reveal the evolution of surface composition (Figure 4c,d and Figure S5, Supporting Information). The valence states of Co in the Co‐20 and Co‐70 samples were dominant by 3+ in the as‐prepared states. After thermal reduction, the appearance of Co^2+^ satellite peak suggests that the Co valence state decreased from 3+ to 2+ (Figure 4c). Such decrease of Co valence state in Co‐20 and Co‐70 samples provides direct evidence for $V_{o}^{••}$ formation,^[^ ^7c^ ^]^ which is in good agreement with lattice expansion we observed in HRXRD results. In comparison to Co, Fe showed less apparent changes in the valence states for all the samples. Such difference was likely due to the lower reducibility of Fe compared to Co, which were also observed previously.^[^ ^34^ ^]^

The Fe 2p spectra for PSFN showed no pronounced difference in peak intensity between the as‐prepared state and after thermal reduction. In contrast, both Fe 2p and Co 2p spectra of the Co‐20 and Co‐70 samples showed weaker intensity and wider width after reduction (Figure 1). Such seemly decrease of XPS intensity was also reported on other perovskite systems such as SrTi_0.3_Fe_0.7_O_3−_ *_*δ*_* ^[^ ^35^ ^]^ and La_0.4_Sr_0.6_FeO_3_^[^ ^36^ ^]^ after surface exsolution as B‐site cation nanoparticles. Due to low concentration of Co‐Fe oxide, no new phase was observed in HRXRD pattern. But B/(A+B) cation ratio provides another strong evidence to support the formation of Co‐Fe enriched nanoparticles. We further evaluated the B/(A+B) cation ratio based on Pr 3d, Sr 3d, Co 2p, Fe 2p, and Nb 3d spectra (Figure 4c,d and Figure S5, Supporting Information). All the tested samples exhibited similar B/(A+B) value in as‐prepared condition (Figure 4e). After reduction, while the B/(A+B) ratio showed negligible changes for the PSFN sample, it decreased abruptly for the Co‐20 and Co‐70 samples. It is widely observed that after thermal reduction B‐site cation exsolved onto perovskite oxide surface and formed nanoparticles.^[^ ^37^ ^]^ The seemly reduction of B/(A+B) ratio we observed here is likely due to limited probing depth of XPS. The probing depths of the Co 2p and Fe 2p XPS spectra were estimated to be less than 3 nm. X ray cannot penetrate the formed B‐site enriched nanoparticles on the reduced Co‐20 and Co‐70 samples, leading to the seemly decreased B‐site content on the surface (Figure 4f).^[^ ^35^, ^36^ ^]^ The reduced Co‐70 sample exhibited the largest exsolved nanoparticles. As a result, it showed the largest decline in B/(A+B) ratio in XPS spectra.

It is difficult to directly map the nanoparticles formed on the thin‐film surface. To confirm the formation of B‐site enriched phase and exsolution on the surface of PSCxFN, we carried out XRD measurement and high‐resolution transmission electron microscope (HRTEM) mapping for the representative powder sample Co‐20 after thermal reduction at 900 °C in 10% H_2_ gas environment, which is a typical reducing condition used in literatures to induce B‐site cation exsolution.^[^ ^21^, ^38^ ^]^ As shown in Figure S6 in the Supporting Information, the exsolved nanoparticles were identified to be Co‐Fe alloy on Co‐20 surface.

It is important to note that we did not observe metallic peak of Co and Fe in the XPS spectra of thin‐film samples (Figure 4c,d). This result suggests that the nanoparticles on the thin films are still Co‐Fe oxide rather than Co‐Fe alloy due to the relatively low reduction temperature.

### 2.5 Electrochemical Performance

**Figure 5:**
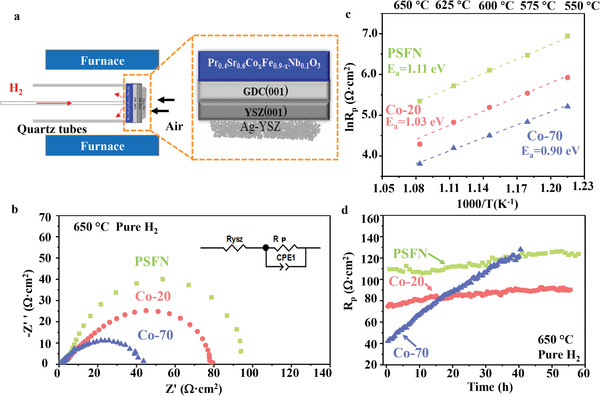
a) Schematic diagram of electrochemical measurement setup for thin‐films systems: PSCxFN thin films as anode in pure H_2_, porous Ag‐YSZ as cathode in air, commercial YSZ substrate as oxygen ion electrolyte. b) Nyquist plots of HOR activity for the PSCxFN thin films at 650 °C. The equivalent circuit used for fitting the EIS data was inserted. c) Temperature dependence of polarization impedance for PSFN (green), Co‐20 (red), Co‐70 (blue) thin film from 650 to 550 °C. The activation energy *E*_a_ calculated from EIS data represents potential energy barrier for HOR. d) Long‐term stability of HOR activity for the PSCFN thin films was investigated at 650 °C.

In this part, we further evaluated the influence of Co doping on the electrochemical performance of PSCxFN. The hydrogen oxidation reaction (HOR) was used as the model reaction to compare the activity and long‐term stability of PSCxFN thin films. The cells with PSCxFN as anode, single‐crystal YSZ substrate as electrolyte and porous Ag‐YSZ as cathode were tested at by using the setup shown in **Figure** **5**a.

The electrochemical impedance spectra (EIS) of the cells were shown in Figure 5b. The spectra were fitted using the equivalent circuit in Figure 5b. The HOR on the PSCxFN thin film anodes made the dominate contribution to polarization resistance (*R*_p_), while the porous cathode showed negligible effect. This result is similar to what we observed in our previous work on thin‐film anodes.^[^ ^7^, ^39^ ^]^ The PSFN exhibited the largest *R*_p_ at 650 °C, indicating its lowest HOR activity (Figure 5b). With Co doping, the *R*_p_ showed the noticeable decrease. Specially, Co‐70 with the highest Co doping level presented the lowest *R*_p_ and fastest HOR kinetics. Such high activity of Co‐70 sample was likely due to the highest $V_{o}^{••}$ concentration and largest amount of Co‐Fe oxide nanoparticles on the surface after thermal reduction. Similar effect of Co doping on HOR activity was also observed at other temperatures in Figure S7 in the Supporting Information.

The activation energy (*E*_a_) of HOR reaction on PSCxFX surface was determined by the Arrhenius plots as shown in Figure 5c. PSFN displayed the largest *R*_p_ value and the highest *E*_a_ of 1.11 eV, suggesting its lowest HOR activity. In contrast, the Co‐70 had the fastest HOR kinetics with the lowest *E*_a_ of 0.90 eV among all the samples. The *E*_a_ of Co‐20 was between that of Co‐70 and PSFN. The decreased *E*_a_ with Co doping in PSCxFN suggests that Co can effectively promote the HOR process on the surface.^[^ ^40^ ^]^

The long‐term stability of PSCxFN was evaluated by carrying out EIS measurement continuously at 650 °C. The polarization of HOR (*R*_p_) on PSCxFN film was plotted as a function of operation time length, as shown in Figure 5d. The PSFN exhibited the highest *R*_p_, but good stability over 55 h operation. The Co‐20 showed the comparable stability with the PSCN samples. In contrast, the *R*_p_ value for the Co‐70 initially was the lowest, but showed the continuous increase over operation time. After 55 h operation, the Co‐70 exhibited noticeably higher *R*_p_ values compared with the PSFN and Co‐20 samples.

The electrochemical measurement above suggests that Co doping can effectively promote the HOR activity of PSCxFN thin films, leading to the highest activity for Co‐70 thin films. The Co doping, nevertheless, also lowers the stability of the thin film, leading to the fastest degradation rate for the Co‐70 thin film.

**Figure 6:**
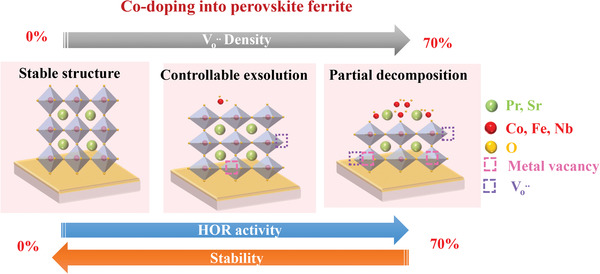
Schematic diagram for evolution of crystal structure and surface B‐site cation behavior after thermal reduction with increasing Co doping in B‐site lattice.

The experimental and computational results shown above consistently suggest that the Co doping has crucial influence on oxygen formation and lattice stability of PSCxFN phase. On one hand, Co doping lowers the oxygen formation energy in PSCxFN, leading to higher density of $V_{o}^{••}$ upon reduction due to the weaker metal—O bonds caused by the increase of antibonding state, and more octahedral distortion of FeO_5_ (**Figure** **6**). On the other hand, the Co doping lowers the lattice stability of PSCxFN, resulting in the easier cation exsolution on the surface. As a result, the PSFN without Co doping exhibited the smallest morphology and surface composition changes, as well as the lowest $V_{o}^{••}$ contents upon hydrogen reduction. In contrast, Co‐70 with highest Co doping showed most pronounced lattice expansion, highest surface $V_{o}^{••}$ concentration, and largest exsolved particles size after reduction. As a consequence of the competing effect of Co doping on the $V_{o}^{••}$ formation and lattice stability, the PSFN exhibited lowest HOR activity but good stability, whereas Co‐70 showed the highest initial HOR activity but poor stability. The Co‐20 sample with moderate Co doping level showed both good HOR activity and stability.

## 3 Conclusion

In this work, highly textured PSCxFN thin films with different Co concentrations at B‐site were prepared by PLD on single‐crystal YSZ substrates as model systems. After thermal reduction in pure H_2_, PSFN exhibited a stable ABO_3_ perovskite structure without obvious XRD peak shift. However, Co‐doping perovskite oxides exhibited larger chemical expansion, which was attributed to the higher content of $V_{o}^{••}$ on the surface. Synchrotron‐based AP‐XPS results further showed that Co‐70 with higher Co content have more $V_{o}^{••}$ formed on surface than that on Co‐20 surface. DFT calculation results suggested that the weaker metal—O bonds caused by the increase of antibonding state as well as more octahedral distortion of FeO_5_ were the main reason of lower $V_{o}^{••}$ formation energy as increased Co doping. As a result of the promoted $V_{o}^{••}$ formation, PSCxFN thin film with higher Co‐doping level possessed larger amount of exsolved particles on the surface. Excessive Co doping until 70% led to immoderate $V_{o}^{••}$ concentration and cation exsolution, which results in partial decomposition of Co‐70. Therefore, its HOR activity deteriorated severely after long‐term operation. In contrast to Co‐70, low content of $V_{o}^{••}$ helped PSFN achieve stable perovskite structure with smooth surface, resulting in good stability but sluggish HOR kinetics. With 20% Co doping, Co‐20 thin film with moderate oxygen defects and exsolved nanoparticles showed good HOR reaction activity and long‐term stability. All the relative results indicate that $V_{o}^{••}$ formation process and structure stability of perovskite oxide can be effectively tuned by bulk doping to enhance electrochemical activity and stability. Our results can provide useful guidance for synthesizing active and stable perovskite oxide electrodes in high‐temperature (electro)chemical devices.

## 4 Experimental Section

### Thin Film Model Systems Synthesis

PSCxFN thin films were deposited on single‐crystal YSZ (CTM wafer) substrate with GDC as buffer layer by PLD using a KrF excimer laser with laser energy of 300 mJ and wavelength of 248 nm. The deposition was carried out at 570 °C under the oxygen pressure of about 1 Pa. The distance from PLD targets to substrates was set to be 6 cm. After deposition, thin‐film samples were cooled down to room temperature with the rate of 5 °C min^−1^ under oxygen pressure of 200 Pa. The synthesis procedure and corresponding XRD pattern (Figure S8, Supporting Information) for the PLD targets can be found in the Supporting information.

### Synchrotron‐Based In Situ Characterization

The surface composition and chemical environment of PSCxFN thin films at elevated temperatures were characterized by using synchrotron‐based AP‐XPS located at Beamline 02B of Shanghai Synchrotron Radiation Facility. X‐ray source and the sample reaction chamber were separated by a SiNx window, which enabled the penetration of X‐ray and protection of X‐ray source. To protect the photoelectron analyzer, which was required to function at high vacuum, a combined differential pump system was equipped to complete the transition from high gas pressure (mbar) in the sample chamber to high vacuum (10^−7^ mbar) in the analyzer chamber. Therefore, the in situ measurement of perovskite thin film with the presence of gas (0.1 mbar for this work) could be realized.^[^ ^41^ ^]^ Before all the characterization, the thin films were heated at 300 °C under 0.1 mbar O_2_ for 1 h to remove carbon and water contamination. Photon energy was set at 1100 eV in this work for measuring Pr 3d, Sr 3d, Co 2p, Fe 2p, Nb 3d, and O 1s core level peaks. Au 4f 7/2 (at 84.0 eV) peak from Au reference sample was used for calibrating the photon energy. All the XPS spectra were collected while the samples were heated at 300 °C in 0.1 mbar O_2_ and 0.1 mbar H_2_ gas environment. The error bar of the cation ratio B/(A+B) in Figure 4e was obtained by fitting XPS data several times, which represented the uncertainty from spectra fitting.

### Ex Situ Material Characterization

XRD (Bruker D8 Advance, Germany) was conducted to determine the crystal structure of PLD targets after preparation. HRXRD (Rigaku SmartLab, Japan) was used to confirm the quality of thin films prepared by PLD. Surface chemistry of thin films was evaluated by using XPS (Thermo Scientific ESCALAB 250Xi) with charge neutralization. To avoid the overlapping between Fe 2p spectra and auger peaks of other elements, Mg K*α* X‐ray radiation was used to detect the Fe 2p peak, while Al K*α* radiation was used for other XPS peaks. The C 1s peak with binding energy of 284.6 eV was used as a reference peak for calibration. The error bar of the cation ratio B/(A+B) in Figure 4e was obtained by fitting XPS date several times, which represented the uncertainty from spectra fitting.

### First‐Principles Calculations

DFT calculations were performed on Pr_0.5_Sr_0.5_Co*_x_* Fe_1−_ *_x_* O_3_ system (PSCxF, *x* = 0, 0.25, 0.50, 0.75) using Vienna ab initio Simulation Package (VASP).^[^ ^42^ ^]^ The electron‐ion interactions were described by using the projector augmented wave potential.^[^ ^43^ ^]^ Generalized gradient approximation (GGA)‐based Perdew–Burke–Ernzerhof functional was used for the exchange‐correlation.^[^ ^44^ ^]^ A plane wave was expanded up to the cutoff energy of 400 eV. Electronic occupancies were calculated by using Gaussian smearing with a smearing parameter of 0.05 eV. For the bulk model of PSCxF series, orthorhombic perovskite structures (space group: *Pmn2_1_*) were used according to the XRD result of PSFN powder. The calculations were performed by using a conjugate gradient algorithm until the forces of each atom were lowered below 0.03 eV Å^−1^ with an energy convergence of 10^−5^ eV. GGA+*U* approach was used to correct the self‐interaction errors with *U*_eff_ = 4.0 eV for Fe 3d orbital and *U*_eff_ = 3.3 eV for Co 3d orbital.^[^ ^45^ ^]^ The oxygen vacancy formation energies and DOS calculations were performed on the slab model with BO_2_‐terminated (1 × 1) surface unit cell, eight‐layer thickness, and vacuum layer of 15 Å. For the slab model, bottom two layers were fixed as in the bulk layers. The COHP calculation was performed with the LOBSTER program and the output of DOS calculation.^[^ ^30^, ^46^ ^]^ For the Brillouin zones of bulk model and slab model, 3 × 3 × 3 and 3 × 3 × 1 Monkhorst−Pack *k*−point samplings were used, respectively.^[^ ^45^ ^]^ Especially, for the DOS calculations, much higher 7 × 7 × 7 Monkhorst−Pack *k*−point samplings were used. The oxygen vacancy formation energy (*E*_f_vac_) was calculated at the surface BO_2_ layers by Equation (1)

(1) $$E_{f\_\mathrm{vac}}=E_{\mathrm{perov}-\mathrm{defect}}+\frac{1}{2}E_{O_{2}}-E_{\mathrm{perov}}$$

where *E*_perov − defect_ and *E*_perov_ are the total energies of slab model with and without an oxygen vacancy, respectively.

The octahedral distortion (Δ) was calculated on the surface half‐octahedron (FeO_5_, CoO_5_). The octahedral distortion (Δ) was calculated by Equation (2)

(2) $$\Delta =\frac{1}{5}\sum {\left(\frac{R_{i}-R_{\mathrm{ave}}}{R_{\mathrm{ave}}}\right)}^{2}$$

where *R_i_* is the bond length of each Fe—O, Co—O bond of the surface half‐octahedron (FeO_5_, CoO_5_) and *R*_ave_ is the average value of five *R_i_*.

### Electrocatalytic Activity Measurement

The HOR performance of PSCxFN thin films was evaluated by constructing an asymmetric cell with the thin film as the anode, GDC as buffer layer, single‐crystal YSZ substrate as the electrolyte, and porous Ag‐YSZ as cathode. The cell was sealed on top of a quartz tube with the PSCxFN film and porous Ag‐YSZ exposing to hydrogen gas and air, respectively. Au pattern collectors were sputtered on the thin‐film surface to serve as the current collector. Zahner Im6 electrochemical station with frequency from 10 kHz to 5 mHz and 10 mV excitation voltage was used for measuring the impedance spectra of the thin‐film cells.

## Conflict of Interest

The authors declare no conflict of interest.

## Supporting information

Supporting Information

## Data Availability

Research data are not shared.
