# Supplementary material for: Activating Lattice Oxygen in Perovskite Oxide by B‐Site Cation Doping for Modulated Stability and Activity at Elevated Temperatures
Source: Adv Sci (Weinh). 2021 Oct 18;8(22):2102713. doi: 10.1002/advs.202102713 (PMC8596113; doi:10.1002/advs.202102713)
Supplement: Supplementary file 1 — Supporting Information [file ADVS-8-2102713-s001.pdf]

© 2021 Wiley-VCH GmbH

ADVANCED SCIENCE
Open Access

Supporting Information

for *Adv. Sci.,* DOI: 10.1002/advs.202102713

Activating lattice oxygen in perovskite oxide by B-site cation doping for modulated stability and activity at elevated temperatures

Huijun Chen, Chaesung Lim, Mengzhen Zhou, Zuyun He, Xiang Sun, Xiaobao Li, Yongjian Ye, Ting Tan, Hui Zhang, Chenghao Yang, Jeong Woo Han * *and Yan Chen* *

Support information

Activating lattice oxygen in perovskite oxide by B-site cation doping for modulated stability and activity at elevated temperatures

Huijun Chen, Chaesung Lim, Mengzhen Zhou, Zuyun He, Xiang Sun, Xiaobao Li, Yongjian Ye, Ting Tan, Hui Zhang, Chenghao Yang, Jeong Woo Han*, Yan Chen*.

1. PLD target synthesis

$Pr_{0.4}Sr_{0.6}Co_xFe_{0.9-x}Nb_{0.1}O_{3-\delta}$ (PSCxFN, x=0, 0.2, 0.7) powders were synthesized by typical combustion method. Ammonium niobate(V) oxalate hydrate, ethylene diamine tetraacetic acid (EDTA) and citric acid with the molar ratio 1 :1 were solved in deionized water at 80 °C, respectively. The temperature was set to be not higher than 90 °C due to the poor thermostability of Ammonium niobate(V) oxalate hydrate. The obtained solutions were mixed with nitrate reagents of metal cations, including stoichiometric amounts of $Pr(NO_3)_3 \cdot 6H_2O$, $Sr(NO_3)_2$, $Co(NO_3)_2 \cdot 6H_2O$, $Fe(NO_3)_3 \cdot 9H_2O$. All these precursors were from Alfa Aesar with high purity (>99%). Ammonium hydroxide was used to adjust the pH of solution to 8. After evaporating at 80 °C overnight, the residues were annealed at 200 °C for the combustion of citric acid to occur. The obtained black precursors were ball-milled, and were subsequently annealed in air at 950 °C for 2 h to form PSCxFN powders. After being grinded for 10 minutes with the addition of PVB-ethanol solution, PSCxFN powders were pressed as a pellet with diameter of about 25 mm and thickness of about 3 mm. The pellets were further sintered at 1300 °C for 6 h to form the PLD targets used in this work.

2. Material Characterization

Surface morphology were characterized by SEM (ZEISS SUPPRA 55VP) and atomic force microscopy (AFM, Asylum Research MFP-3D-S).

3. Chemical Expansion

The changes in lattice parameter introduced by the formation of oxygen defects, [1] as shown by following equation (**Equation S1**)

$$\varepsilon = \frac{a - a_0}{a_0} \quad \textbf{(S1)}$$

in which the lattice parameter a and a_o are determined by HRXRD for the films in the as-prep. state and after the generation of oxygen vacancies in the lattice through thermal reduction at 650 °C.

4. Activation Energy (E_a)

The temperature dependence of electrical conductivity through the small polaron hopping mechanism is expressed by:

$$\sigma = \frac{A}{T} \exp\left(\frac{-E_a}{kT}\right) \ldots\ldots\ldots\ldots\ldots\ldots\ldots\ldots\ldots\textbf{(S2)}$$

Where E_a represents activation energy, T stands for the absolute temperature, A is a pre-exponential factor, and k id the Boltzmann constant[2]. The slope of the log σT versus 1/T line gives the values of E_a, which calculated from the plots of the Arrhenius plot in Figure. 4 by linear regression.

5. Results

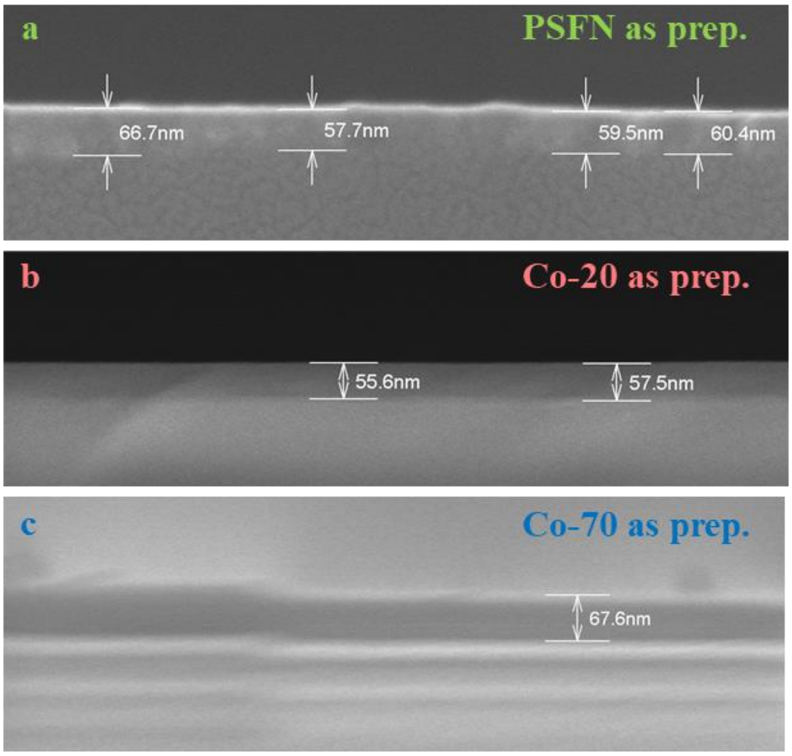

Figure S1. SEM images for cross section of thin film systems after PLD deposition process: a) PSFN, b) Co-20 and c) Co-70.

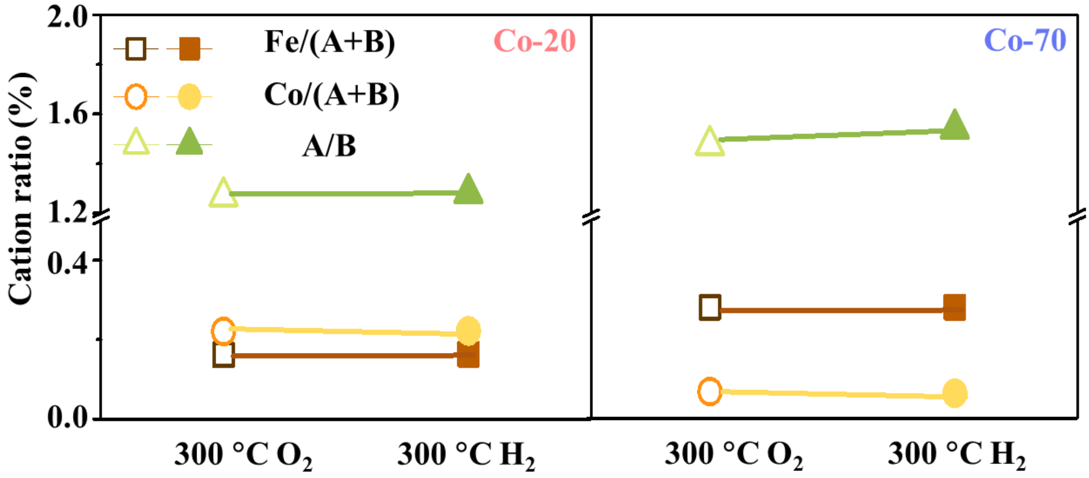

Figure S2. Cation ratios of Fe/(A+B), Co/(A+B) and A/B of thin film systems Co-20 (left) and Co-70 (right) after annealed at 300 °C in different gas atmospheres, including O_2 and H_2.

Table S1. Summary of fitting parameters for O 1s peak, including area, position for Co-20 and Co-70 treated at 300 °C in O_2 or H_2 in the AP-XPS chamber.

**Samples**	**Oxygen species**	**Peak position**	**Peak area (300 °C O_2)**	**Peak area (300 °C H_2)**
	surface O	530.9	11.8	11.7
Co-20	highly oxidative O	529.6	7.1	7.1
	lattice O	528.5	69.9	67.9
	surface O	530.9	13.7	16.9
Co-70	highly oxidative O	529.6	5.3	5.8
	lattice O	528.5	50.6	50.5

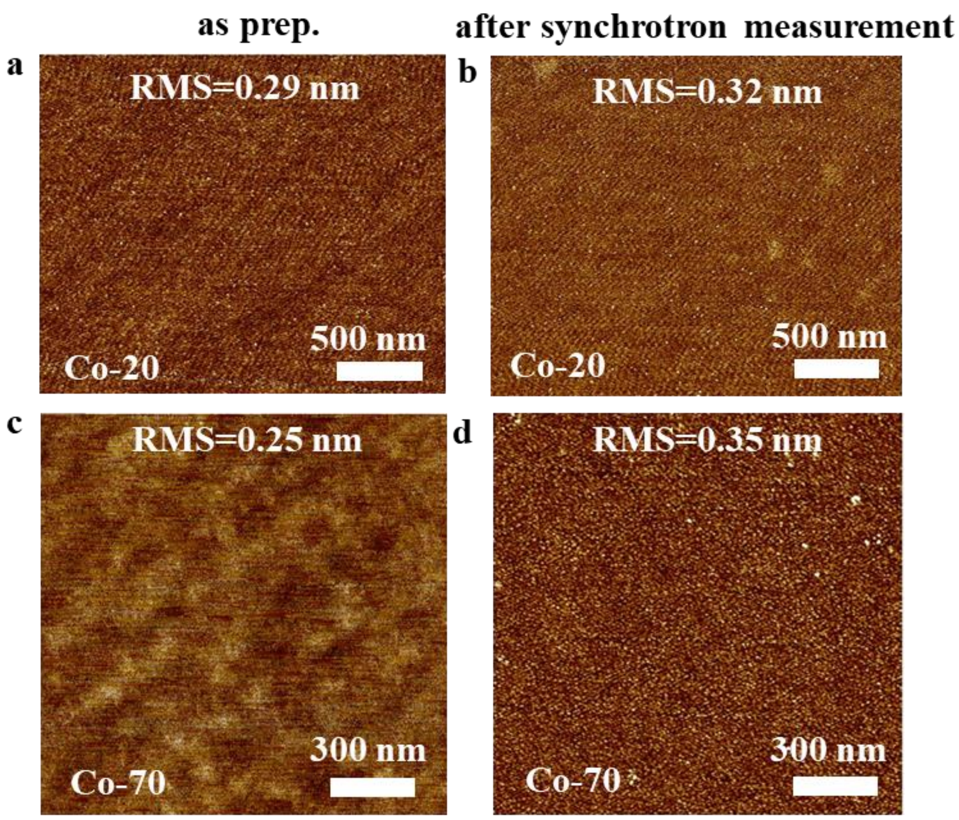

Figure S3. Surface morphologies and corresponding roughnesses of a-b) Co-20 or c-d) Co-70/GDC/YSZ in their as prep. condition and after synchrotron experiment.

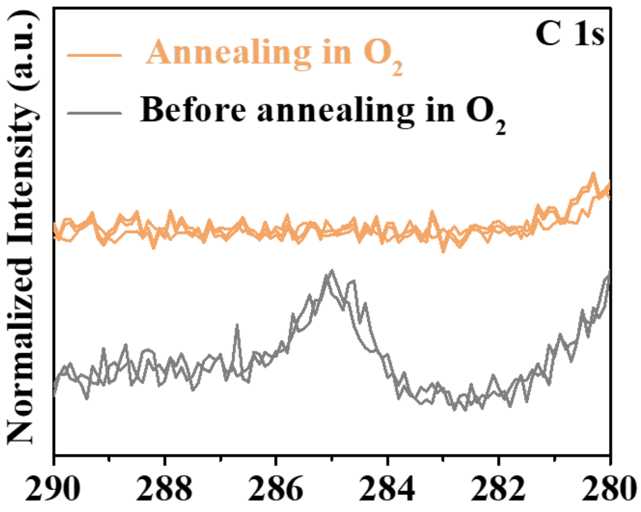

Figure S4. C 1s spectra were detected in as prep. condition and after annealed in 0.1 mbar O_2 for 1 h.

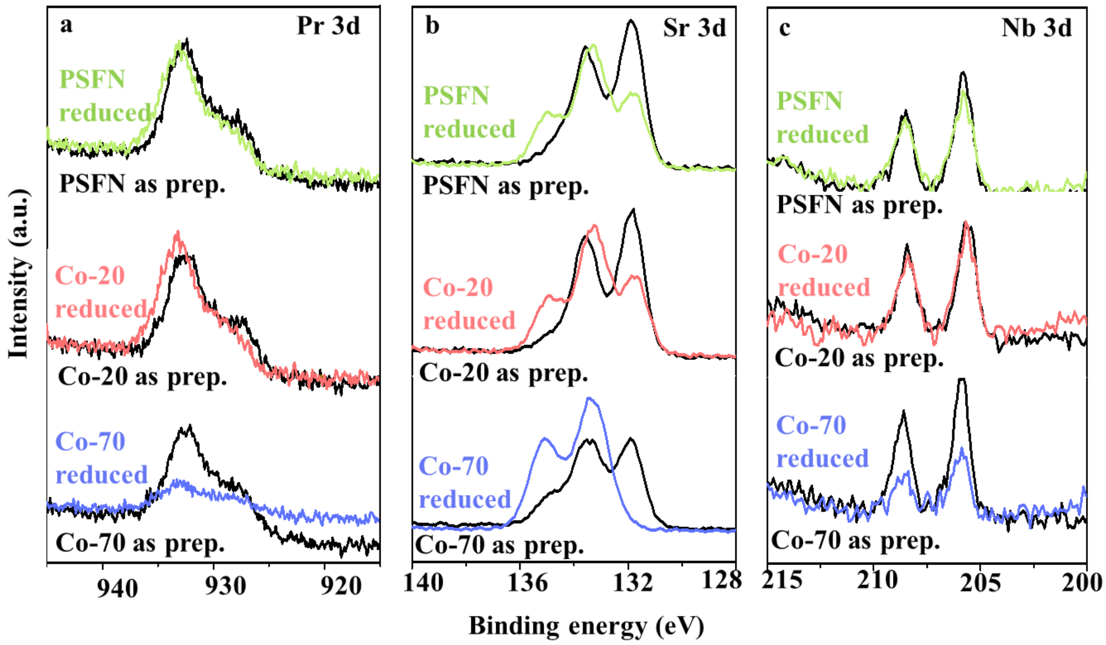

Figure S5. XPS spectra for PSCxFN thin films in their as-prepared state (bottom) and after reduced (top), including a) Pr 3d, b) Sr 3d and c) Nb 3d.

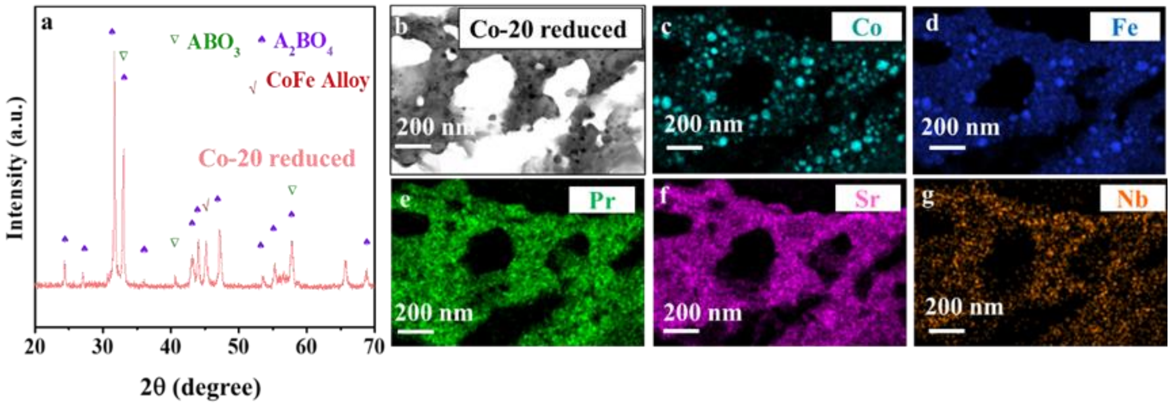

Figure S6. a) XRD pattern, (b) Transmission electron microscopy (TEM) images, and EDS elemental maps of c) Co, d) Fe, e) Pr, f) Sr and g) Nb for the Co-20 powder synthesis by critic combustion method after reduced in 900 °C for 2 h in 10% H_2.

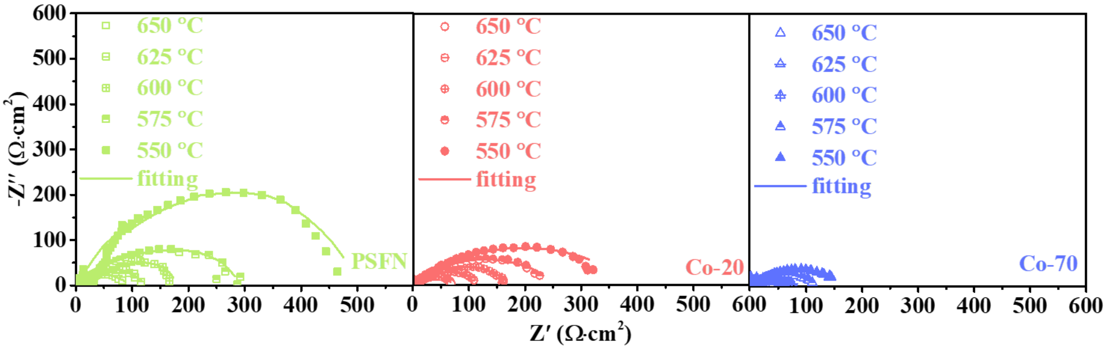

Figure S7. Nyquist plot for PSCxFN thin films at different temperatures with range from 650 °C to 550 °C with pure H_2 as fuel gas: a) PSFN, b) Co-20 and c) Co-70.

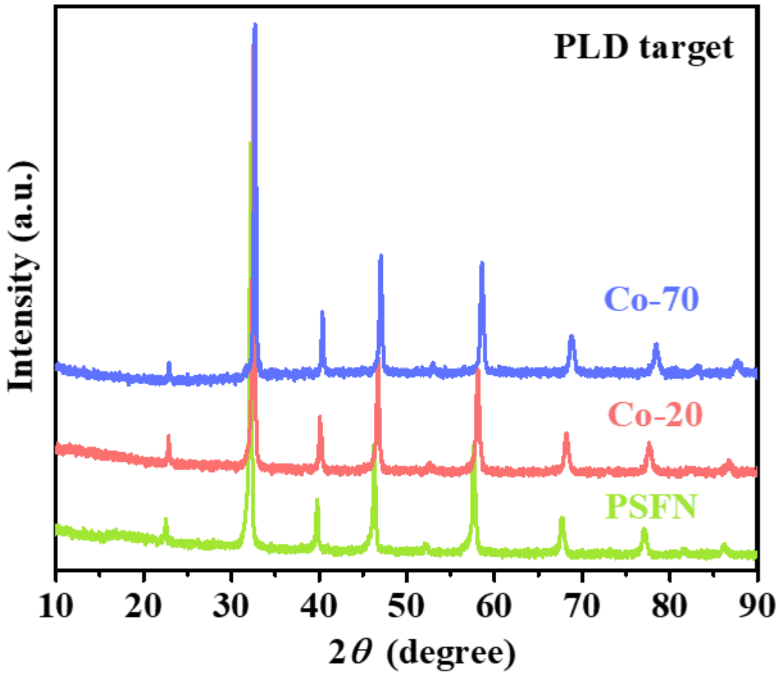

Figure S8. XRD patterns for PLD targets: PSFN, Co-20 and Co-70.

[1]Marrocchelli D., Bishop S.R., Tuller H.L., et al. Understanding chemical expansion in non-stoichiometric oxides: ceria and zirconia case studies[J]. Advanced Functional Materials, 2012, 22 (9): 1958-1965.

[2]Ding X., Gao Z., Ding D., et al. Cation deficiency enabled fast oxygen reduction reaction for a novel SOFC cathode with promoted CO2 tolerance[J]. Applied Catalysis B: Environmental, 2019, 243: 546-555.
